# Common dietary patterns and risk of cancers of the colon and rectum: Analysis from the United Kingdom Women's Cohort Study (UKWCS)

**DOI:** 10.1002/ijc.31362

**Published:** 2018-04-01

**Authors:** Diego Rada‐Fernandez de Jauregui, Charlotte E.L. Evans, Petra Jones, Darren C. Greenwood, Neil Hancock, Janet E. Cade

**Affiliations:** ^1^ Preventive Medicine and Public Health Department University of the Basque Country/Euskal Herriko Unibertsitatea (UPV/EHU) Vitoria‐Gasteiz Spain; ^2^ Nutritional Epidemiology Group, School of Food Science and Nutrition, University of Leeds Leeds United Kingdom; ^3^ Department of Food Sciences and Nutrition University of Malta Msida Malta; ^4^ Division of Epidemiology and Biostatistics School of Medicine, University of Leeds Leeds United Kingdom; ^5^ Leeds Institute of Data Analytics, University of Leeds Leeds United Kingdom

**Keywords:** cohort study, dietary patterns, red meat, poultry, fish, vegetarian, colonic neoplasm, rectal neoplasm and epidemiology

## Abstract

Few prospective cohort studies in the UK have specifically focused on the associations between commonly consumed dietary patterns and colorectal cancer (CRC). The aim of our study was to assess whether red meat, poultry, fish and vegetarian dietary patterns are associated with differences in the incidence of cancers of colon and rectum in the UKWCS. Four common dietary patterns were defined based on a hierarchy of consumption of red meat, poultry and fish for each cohort participant, using a 217‐item food frequency questionnaire. Cox proportional hazards regression was used to provide adjusted hazard ratios (HR) and 95% confidence intervals (CI) for CRC. A total of 32,147 women recruited and surveyed between 1995 and 1998 were followed up for a mean of 17.2 years (426,798 person‐years). A total of 462 incident CRC cases were documented; 335 colon cancers (172 proximal and 119 distal) and 152 in the rectum. In multivariable‐adjusted models, there was no evidence of a reduction in risk of overall CRC (HR = 0.86, 95% CI: 0.66–1.12), colon cancer (HR = 0.77, 95% CI: 0.56–1.05) or rectal cancer (HR = 1.04, 95% CI: 0.66–1.63) when comparing grouped red meat free diets with diets containing red meat. Exploratory analysis suggested a reduced risk of distal colon cancer in grouped red meat free diets (HR = 0.56, 95% CI: 0.34–0.95), though numbers with this outcome were small. These results indicate that a protective association of red meat free diets specifically on distal colon cancer merits confirmation in a larger study.

## Abstract 2

**What's new?**

Eating red meat may affect one's risk of colon cancer. Here, the authors looked for a relationship between colorectal cancer risk and red meat, poultry, fish, and vegetarian diets. They used data from the UK Womens Cohort study, with dietary information collected by survey. No statistically significant association was found for total CRC risk, but when they looked at specific cancer sub‐sites, a correlation emerged. Although numbers were small, a reduction in distal CRC risk was observed among those who did not eat red meat. A larger study would be desirable to confirm these findings.

**Abbreviations**
CI: confidence intervals
CRC: colorectal cancer
EPIC: investigation into cancer
FFQ: food frequency questionnaire
HR: hazard ratios
IARC: International Agency for Research on Cancer
SES: socio‐economic status
UKWCS: UK Women's Cohort Study
WCRF: World Cancer Research Fund

## Introduction

Colorectal cancer (CRC) is the second most commonly diagnosed cancer in women and the third in men worldwide.^1^ The International Agency for Research on Cancer (IARC) in 2015 classified red meat as “probably carcinogenic to humans” and processed meat as “carcinogenic to humans,” based mainly on evidence linked to CRC.^2^ The most recent (September 2017), World Cancer Research Fund (WCRF) and American Institute for Cancer Research (AICR) continuous update project (CUP) have arrived to no conclusion due to limited evidence on dietary patterns and CRC.^3^ Different meta‐analyses indicate that high intake of red meat and processed meat is associated with significant increased risk of colorectal, colon and rectal cancer.^4^, ^5^, ^6^, ^7^ Hence, vegetarian diets or low meat diets may be expected to be associated with a lower risk of CRC given their lack of, or reduced, meat content, but current scientific evidence remains inconsistent and requires further explanation. Some of the inconsistency in findings may be owing to the complete exclusion of any source of meat or fish protein from the diet (pure vegetarian diet)^8^ and CRC subsites.^9^ Early results from the EPIC‐Oxford study found an approximately 50% greater risk of CRC for vegetarians.^10^ Later, as incident cases increased, adverse associations for vegetarians turned into null, in regard to both CRC mortality^11^, ^12^ and CRC incidence.^13^, ^14^ Analysis from pooled data from prospective food diaries, among UK cohorts with low to moderate meat intakes, showed little evidence of association between consumption of red and processed meat and CRC risk.^15^ However, the prospective cohort trial of Seventh Day Adventist in the USA has found that vegetarian diets, especially pesco‐vegetarians, those who eat fish but no meat, are associated with an overall lower incidence of CRC.^16^ Recently, fish intake has also been found to be inversely associated with the risk of rectal cancer.^17^ Another cohort study carried out in the Netherlands found pesco‐vegetarians and 1 day/week meat eaters had a modest but non‐significantly decreased risk of CRC compared to 6–7 day/week meat consumers.^18^ The traditional approach in nutritional epidemiology concentrates on the effects of single nutrients or foods on CRC. However, nutrients and foods are consumed in combination, so effects on disease risk benefit from considering the entire eating pattern. Dietary patterns may go further than individual nutrient exposures when explaining disease occurrence^19^ and can be easier to translate into public health recommendations compared to focusing on individual nutrients.^20^

The UK Women’ s Cohort Study (UKWCS) is a large British cohort of women with a long follow up period and was designed to include a wide range of different meat and meat‐free dietary intakes. Here, we examine the associations between common dietary patterns including red meat eaters, poultry eaters, fish eaters and vegetarians and the association with the incidence of cancers of the colon and rectum. An exploratory analysis of the risk of colon cancer subsites is also presented.

## Methods

### Study design, study population and ethical approval

Women were recruited into the UKWCS from responders to a direct mail survey of the World Cancer Research Fund (WCRF) between 1995 and 1998, with around half a million responders from England, Wales and Scotland. Further details of the process have been described previously.^21^ The WCRF questionnaire included brief dietary details allowing selection of all women who characterized themselves as vegetarian or non‐red‐meat eaters and a comparison group from the remaining eligible women. The comparison group was chosen by matching by age, within 10 years of each vegetarian, to the next non‐vegetarian responder. A total of 35,372 women aged 35–69 years returned the baseline postal questionnaire. A specific feature of the UKWCS was that it was designed to include large numbers of subjects consuming three main dietary patterns: vegetarian, eating fish (not meat) and meat eaters.^22^ This approach was adopted to maximize power for comparisons of interest between diet and cancer while minimizing the effect of measurement error.^23^, ^24^, ^25^ Ethical approval was granted at its initiation in 1993 (Research Ethics Committee reference number is 15/YH/0027).

### Baseline characteristics and dietary patterns construction

Anthropometrics, lifestyle factors and socio‐demographic information were self‐reported with socio‐economic status (SES) based on occupation. Information on physical activity was collected by questionnaire. The participants’ diet was assessed using a 217‐item, self‐administered food frequency questionnaire (FFQ). The FFQ was based on that used in the Oxford arm of the Investigation into Cancer (EPIC) study and adapted for use with vegetarians. Completion of the questionnaire simply required placing a tick in the box to indicate how frequently each food had been consumed over the last 12 months. Any single missing items were assumed to have not been consumed. Standard portion weights were assigned and energy and nutrient intakes was derived using McCance & Widdowson's *The Composition of Foods* (5th Edition) (Holland *et al*., 1991). In this analysis, four commonly recognized eating patterns were used based on response frequencies of meat and fish items on the FFQ. Vegetarians were defined as those participants who consumed red meat poultry, or fish less than once a week; fish eaters were defined as those participants who consumed fish at least once a week but not poultry or red meat; poultry eaters were defined as those participants who consumed poultry at least once a week and may eat fish but not red meat; and red meat eaters were defined as those participants who consumed meat at least once a week and may or may not consume poultry and fish. Red meat is defined as beef, pork, lamb, offal and processed meats.^26^

### Case definition

Registrations of cancer diagnosis for women in the UKWCS were made via record linkage of cancer identification codes from the central register of NHS Digital. The cancer outcomes used in the analyses are incident malignant neoplasms of the colon (codes 153.0–153.9 or C18) and of the recto sigmoid junction and of the rectum (codes 154.0–154.1 or C19 and C20) of the International Statistical Classification of Diseases (ICD), 9th and 10th editions.^27^, ^28^ Cancer of the colon included proximal colon tumors (cecum, appendix, ascending colon, hepatic flexure, transverse colon and splenic flexure: C18.0–C18.5) and distal colon tumors (descending and sigmoid colon: C18.6 and C18.7). Colon cancers were defined in the ICD as those occurring above the peritoneal delineation of the abdominal cavity, and rectal cancers were those occurring below this delineation. Tumors originating proximal to the splenic flexure (cecum, ascending colon and transverse colon) were considered proximal colon cancers, whereas those tumors arising in the descending or sigmoid colon were considered distal colon cancers. Recto sigmoid cancers were defined as rectal cancers and anal cancers were excluded from the analysis as described in previous publications.^29^

### Statistical analysis

Descriptive statistics were used to describe baseline characteristics of participants according to dietary patterns. Survival analysis was conducted to explore the relationship between four dietary patterns and colorectal, colon (exploratory analysis of proximal and distal colon subsites) and rectal cancer risk. Cox proportional hazards regression was used to provide hazard ratios (HRs) and 95% confidence intervals (CI) for the estimation of relative risk of cancers. The red meat eating category was used as reference category. The time variable used in the models was time in the study, calculated from the date of questionnaire receipt until either death or censor date (1st of April 2014). Covariates were selected for inclusion in the regression models based on published information on convincing confounders for CRC. Associations were estimated first as a simple age‐adjusted model, and finally as a multivariable adjusted model including age (years), body mass index (BMI) (kg/m^2^), energy intake (kcal/day), physical activity (hr/day), smoking status (never, current or former smoker), family history of CRC in a first degree relative and socio‐economic status (professional/managerial, intermediate or routine and manual). Education was not included because too many women were lost due to the missing data and also because it is potentially correlated with socioeconomic status. As a sensitivity analysis other nutritional variables such as ethanol consumption, dietary fiber, calcium, iron and folate and risk factors like polyps in the large intestine were included as additional confounders but no substantial differences were observed in the results (data not shown). Further analysis of robustness of results was carried out by merging the poultry and fish eaters into one group due the low number in the poultry group. The proportional hazards assumption was tested graphically for all terms in the model. To account for the stratified sampling scheme at recruitment, over‐sampling vegetarians and fish‐eaters, statistical models used weights based on the inverse probability of being sampled to provide estimates more representative of the UK population.^26^, ^29^ All the statistical analyses were conducted using Stata version 13 statistical software.^30^

## Results

### Baseline characteristics according to dietary pattern

Of 35,372 women available at baseline, we excluded women who did not provide sufficient data at baseline to allow flagging for cancer incidence notification on NHS Digital (*n* = 688), women self‐reporting history of any previous malignant cancer at baseline, except for non‐melanoma of the skin (*n* = 2,398), women who were diagnosed with CRC within 1 year of baseline (*n* = 53) and women with energy intakes outside the plausible range of 500–6,000 kcal/day (*n* = 86). After these exclusions, 32,147 cohort participants were eligible for this analysis. Of these, 65% (20,848) were classified as red meat eaters, 3% (899) were poultry eaters, 13% (4,141) were fish eaters and 19% (6,259) were vegetarians. Some demographic and lifestyle characteristics, medical history, as well as nutrient and food intake at baseline data collection of these groups are summarized in Table 1. At baseline, the mean age was 52 years and the average BMI 24.4 (kg/m^2^). Cohort participants were relatively health conscious, with a low proportion of smokers (11%) and a large proportion reported taking dietary supplements (58%). More detail regarding the UKWCS cohort has been reported previously.^21^, ^22^ Women in the poultry eaters, fish eaters and vegetarian groups were likely to be younger, had a lower BMI and engaged in more physical activity compared to red meat eaters. Physical activity was highest in the fish eaters and lowest in the red meat eaters. A higher percentage of the fish eaters and vegetarians were from a professional and managerial social background compared to the red meat and poultry eating groups. Self‐reported history of polyps in the large intestine was higher in the red and poultry groups with fairly similar history of CRC in four groups. Red meat eaters and fish eaters tended to have a higher energy intake and higher alcohol intake and fish eaters had highest consumption of fiber, iron, calcium, folate and vitamin C. To account for these differences observed, we controlled for the corresponding variables in multivariate analyses.

**Table 1:** Baseline characteristics according to four common dietary patterns from the UKWCS

	Dietary pattern type				
Variable	Red meat eater	Poultry eaters	Fish eaters	Vegetarians	Total
Demographic characteristics					
N (%)	20,848 (64.9)	899 (2.8)	4,141 (12.9)	6,259 (19.5)	32,147
Age (yr), mean (SD)	53.3 (9.3)	52.5 (9.2)	50.5 (8.9)	48.6 (8.4)	52.02 (9.3)
BMI (kg/m2), mean (SD)	25.0 (4.4)	23.6 (3.8)	23.3 (3.4)	23.4 (3.9)	24.4 (4.2)
Professional/managerial SES, N (%)	12,248 (60.1)	538 (61.2)	2,885 (71.0)	4,281 (70.0)	19,952 (63.4)
Degree level of education, N (%)	4,308 (22.8)	183 (22.2)	1,373 (35.6)	2,208 (37.3)	8,073 (27.4)
Lifestyle characteristics					
Physical activity (hr/day), mean (SD)	0.23 (0.47)	0.27 (0.51)	0.30 (0.54)	0.28 (0.47)	0.25 (0.49)
Current smoker, N (%)	2,366 (11.7)	79 (9.1)	412 (10.3)	626 (10.3)	3,483 (11.2)
Supplement users, N (%)	10,185 (53.6)	576 (71.7)	2,511 (67.2)	3,540 (62.2)	16,812 (57.5)
Medical history					
Polyps in large intestine, N (%)	194 (1.02)	10 (1.2)	25 (0.6)	27 (0.5)	256 (0.9)
Family history of colorectal cancer, N (%)	1,225 (6.2)	55 (6.5)	227 (6.0)	323 (5.5)	1,830 (6.0)
Energy and nutrient intake					
Energy intake (kcal/day), mean (SD)	2,293(703)	2,255(724)	2,293(727)	2,220(720)	2,278(711)
Fat intake (g/day), mean (SD)	85.9 (31.8)	77.6 (30.6)	77.6 (30.6)	81.4 (33.3)	84.5 (32.3)
Saturated fat intake (g/day), mean (SD)	30.7 (13.3)	25.3 (11.8)	25.3 (11.8)	26.4 (13.0)	29.3 (13.3)
Non milk extrinisic sugar intake (g/day), mean (SD)	84.0 (44.3)	82.1 (47.0)	82.1 (47.0)	77.7 (41.3)	82.3 (43.6)
Fiber intake (g/day), mean (SD)	23.8 (9.4)	27.8 (11.7)	28.7 (10.9)	28.4 (11.1)	25.4 (10.3)
Iron (mg/day), mean (SD)	17.9 (7.2)	18.6 (7.7)	18.9 (7.8)	18.1 (7.4)	18.1 (7.4)
Calcium (mg/day), mean (SD)	1,139 (366)	1,156 (416)	1,202 (422)	1,139 (413)	1,147 (385)
Folate (μg/day), mean (SD)	392.6 (127.6)	420.0 (154.4)	421.7 (145.6)	413.0 (144.8)	401.1 (134.8)
Vitamin C (mg/day), mean (SD)	167.7 (81.4)	188.1 (102.9)	185.0 (92.6)	178.3 (93.8)	172.6 (86.4)
Food intake					
Portions of fruit and vegetables (no./day), mean (SD)	3.0 (1.4)	3.3 (1.6)	3.4 (1.6)	3.4 (1.7)	3.1 (1.5)
Ethanol (g/day), mean (SD)	9.1 (10.8)	7.4 (9.3)	8.8 (10.2)	7.4 (10.1)	8.7 (10.6)
Red meat consumption (g/day), mean (SD)	51.6 (38.4)	1.7 (3.2)	0.4 (1.6)	0.1 (0.8)	33.5 (39.5)
Processed meat consumption (g/day), mean (SD)	19.1 (14.6)	1.3 (1.8)	0.4 (1.1)	0.1 (0.5)	12.5 (14.8)
Poultry consumption (g/day), mean (SD)	24.0 (19.6)	39.7 (27.5)	1.9 (3.8)	0.3 (1.5)	16.9 (20.0)
Fish consumption (g/day), mean (SD)	32.0 (23.2)	46.0 (32.9)	43.3 (31.1)	1.8 (3.4)	28.0 (26.2)

Abbrevitions: SD: standard deviation; SES: socio‐economic status.

### Red meat and red meat‐free dietary patterns

Over a mean follow up of 17.2 years a total of 462 incident CRC cases were documented in the UKWCS (426,798 person‐year). Of these cases, 335 were colon cancers, 172 in the proximal and 119 in the distal colon, and 152 in the rectum; 25 cases were diagnosed with both colon and rectal cancer and information on subsite was not available for 44 colon cancers. Table 2 presents the results of Cox proportional hazards regression models for grouped red meat‐free diets compared to red meat diet, for all colorectal cancers combined, colon (combined and subsites) and rectal cancers, separately. Red meat‐free diets showed a non‐significant risk reduction in overall CRC (HR = 0.86, 95% CI: 0.66–1.12) and colon cancer (HR = 0.77, 95% CI: 0.56–1.05) with risk close to the null in the case of the rectal cancer (HR = 1.04, 95% CI: 0.66–1.63). In the exploratory analysis of colon subsites a significant risk reduction on distal colon cancer was observed in red meat‐free diets compared to red meat diets in both the age adjusted model (HR = 0.58, 95% CI: 0.36–0.92) and the fully adjusted model (HR = 0.56, 95% CI: 0.34–0.95).

**Table 2:** Hazard ratios (95% confident interval) for risk of colorectal cancer, including all subsites according to red meat and red meat free diets (*n* = 32,147)

Cancer site	Diettary pattern	Cases/noncases	Age‐adjusted HR	95% CI	Multivariable‐adjusted HR^a^	95% CI
Colorectal						
	Red meat diet	399/25,485	1	Ref	1	Ref
	Red meat free diet	63/6,196	0.81	0.64–1.02	0.86	0.66–1.12
Test for difference between groups			0.074		0.254	
Colon						
	Red meat diet	296/25,592	1	Ref	1	Ref
	Red meat free diet	39/6,220	0.7	0.52–0.92	0.77	0.56–1.05
Test for difference between groups			0.012		0.100	
Proximal colon						
	Red meat diet	153/25,735	1	Ref	1	Ref
	Red meat free diet	19/6,240	0.80	0.54–1.19	0.87	0.56–1.35
Test for difference between groups			0.265		0.525	
Distal colon						
	Red meat diet	101/25,787	1	Ref	1	Ref
	Red meat free diet	18/6,241	0.58	0.36–0.92	0.56	0.34–0.95
Test for difference between groups			0.021		0.032	
Rectal						
	Red meat diet	126/25,762	1	Ref	1	Ref
	Red meat free diet	26/6,233	1.07	0.72–1.60	1.04	0.66–1.63
Test for difference between groups			0.734		0.865	

a Adjusted for age (years), body mass index (BMI) (kg/m^2^), energy intake (kcal/day), physical activity (hr/day), smoking status (never, current or former smoker), family history of CRC in a first degree relative and socio‐economic status (professional/managerial, intermediate or routine and manual).

In the sensitivity analysis where poultry and fish eaters were merged, estimates were broadly similar and conclusions did not change. These two types of dietary patterns tend to have a similar effect on risk of CRC.

### Dietary patterns and colorectal cancers

The association between the common dietary patterns and risk of CRC, both overall and by subsites (exploratory), are presented in Table 3, for both Model 1 (age‐adjusted) and Model 2 (multivariable‐adjusted). In multivariable‐adjusted models, poultry eaters (HR = 0.85, 95% CI: 0.45–1.60), fish eaters (HR = 0.90, 95% CI: 0.63–1.29) and vegetarians (HR = 0.80, 95% CI: 0.58–1.11) showed a non‐significant risk reduction compared to red meat eaters for risk of CRC. In the fully adjusted model, vegetarians showed the highest risk reduction compared to red meat eaters in the case of colon cancer, although confidence intervals were wide (HR = 0.71, 95% CI: 0.47–1.08). In a sensitivity analysis to study the impact of high (≥ 130 grams per day) and low/medium read meat and processed meat consumption (<130 grams per day) on CRC, again, low/moderate red meat eaters (HR = 0.84, 95% CI: 0.57–1.25), and the other three red meat free patterns (poultry eaters, fish eaters and vegetarians) showed a non‐significant risk reduction (*p* = 0.062) compared to high red meat eaters for risk of CRC in multivariable‐adjusted models.

**Table 3:** Hazard ratios (95% confident interval) for risk of colorectal cancer, including all subsites according to four common dietary patterns (*n* = 32,147)

Cancer site	Diettary pattern	Cases/noncases	Age‐adjusted HR	95% CI	Multivariable‐adjusted HR^a^	95% CI
Colorectal						
	Red meat eater	342/20,506	1	Ref	1	Ref
	Poultry eaters	13/886	0.88	0.51–1.53	0.85	0.45–1.60
	Fish eaters	44/4,097	0.75	0.54–1.05	0.90	0.63–1.29
	Vegetarians	63/6,196	0.83	0.61–1.13	0.80	0.58–1.11
Test for difference between groups			0.280		0.572	
Colon						
	Red meat eater	257/20,591	1	Ref	1	Ref
	Poultry eaters	5/894	0.45	0.19–1.08	0.57	0.24–1.38
	Fish eaters	34/4,107	0.78	0.53–1.14	0.91	0.60–1.38
	Vegetarians	39/6,220	0.75	0.50–1.11	0.71	0.47–1.08
Test for difference between groups			0.100		0.267	
Proximal colon						
	Red meat eater	130/20,718	1	Ref	1	Ref
	Poultry eaters	4/895	0.7	0.26–1.87	0.88	0.32–2.36
	Fish eaters	19/4,122	0.87	0.52–1.46	0.96	0.54–1.72
	Vegetarians	19/6,240	0.77	0.43–1.38	0.73	0.39–1.37
Test for difference between groups			0.713		0.802	
Distal colon						
	Red meat eater	91/20,757	1	Ref	1	Ref
	Poultry eaters	1/898	0.26	0.04–1.84	0.33	0.05–2.37
	Fish eaters	9/4,132	0.48	0.24–0.96	0.55	0.26–1.15
	Vegetarians	18/6,241	0.91	0.51–1.62	0.74	0.40–1.36
Test for difference between groups			0.111		0.248	
Rectal						
	Red meat eater	104/20,744	1	Ref	1	Ref
	Poultry eaters	8/891	1.74	0.84–3.59	1.37	0.55–3.41
	Fish eaters	14/4,127	0.79	0.43–1.44	0.98	0.52–1.85
	Vegetarians	26/6,233	1.03	0.63–1.66	0.91	0.55–1.52
Test for difference between groups			0.385		0.883	

a Adjusted for age (years), body mass index (BMI) (kg/m^2^), energy intake (kcal/day), physical activity (hr/day), smoking status (never, current or former smoker), family history of CRC in a first degree relative and socio‐economic status (professional/managerial, intermediate or routine and manual).

In the case of rectal cancer, vegetarians (HR = 0.91, 95% CI: 0.55–1.52) and fish eaters (HR = 0.98, 95% CI: 0.52–1.85) were close to the null effect while the small group of poultry eaters showed an increased risk of rectal cancer but as with previous results, intervals were very wide CI (HR = 1.37, 95% CI: 0.55–3.41). Exploratory analysis between the dietary patterns and the different colon subsites cancer risk showed broadly similar associations but with higher effect sizes in the case of distal colon (Table 3). Poultry eaters (HR = 0.33, 95% CI: 0.05–2.37) showed the highest associated risk reduction but the 95% CI was wide.

## Discussion

UKWCS is a large cohort with varied dietary intakes, including a high number of non‐red‐meat eaters, and a long follow up period; consequently this is one of the largest analyses comparing commonly consumed dietary patterns and the risk of CRC in the UK. In our study, there was insufficient evidence for any differences between the dietary pattern groups and risk of CRC, though confidence intervals were wide. In the UKWCS, red meat eaters were more likely to be older and less well educated^11^ with a higher body mass index and lower physical activity and fruit and vegetable intake than the other groups. A similar pattern was seen in participants who were most likely to eat meat in the EPIC cohort.^31^ The red meat eating dietary pattern in our cohort consumed on average relatively low amounts of red meat (mean 51.6 g/day) and processed meat (mean 19.1 g/day). Our results are not statistically significant for red meat eating dietary patterns and overall risk of CRC, however, a red meat‐free diet was significantly protective against distal colon cancer. This is of interest from a public health point of view as in this cohort, a red meat eating pattern characterized by lower overall meat intakes, may be generally at lower risk of colorectal cancers compared to populations with a higher meat consumption; for example, women aged 35–59 years in the National Diet and Nutrition Survey are consuming on average 131 g meat/Day.^32^ There is a biological plausibility of the effect of red meat on CRC related mainly with components in red meat or which are formed during the cooking of meat that may increase colorectal cancer risk including animal fat, heme iron, heterocyclic amines (HCAs) and the endogenous formation of N‐nitroso compounds (NOCs),^33^ but dose–response relationships and specially the effect of low meat intake on CRC remains unclear.^4^ In a sensitivity analysis comparing high (≥130 g per day) and low/medium read meat and processed meat consumption (<130 g per day) on CRC we did not find a significant risk reduction for the risk of CRC.

Additional foods in the diet other than red meat may be also associated with a decreased risk of CRC including milk and whole grains.^34^, ^35^ Specific nutrients such as calcium and fiber which are present in high levels in those foods, have also been associated with a lower risk of CRC.^34^ Analysis based on nutrient patterns suggests that patterns characterized by high intakes of vitamins and minerals are inversely associated with CRC as is a pattern rich in riboflavin, phosphorus and calcium.^36^ In our analysis we explored differences in dietary variables (calcium, folate and fiber) between dietary patterns (data not shown) but did not see substantial differences between patterns. For this reason they were not included in the model to avoid over adjusting.^37^ Recent studies have found that particularly pesco‐vegetarians were at lower risk of CRC.^16^, ^17^ We observed a possible protective association between fish and vegetarian diets and subsequent CRC incidence but as in previous UK‐based studies^11^, ^12^, ^13^, ^14^ no statistically significant differences were observed. Fish eaters in the UKWCS were younger, with a lower energy intake, and were more likely to consume 400 g or more of fruit and vegetables per day. A recent review suggests that data on Selenium (Se) intake and status in British vegetarians could help to explain why studies on vegetarians in the UK present different results from the US. British vegetarians may be more likely to have a low Se status and this may contribute to the largely null results of studies of CRC risk in vegetarians in the UK.^2^ In concordance, results from a case–control study of the EPIC cohort indicates that Se status is suboptimal in many Europeans and suggests an inverse association between CRC risk and higher serum Se status, especially in women^38^ In our study, the null results can be also explained because our definition of vegetarian was not completely strict, allowing vegetarians to consume meat, poultry or fish in small amounts (less than once a week). Existing evidence that *n*‐3 fatty acids inhibit colorectal carcinogenesis is in line with these results, but few data are available addressing this association.^39^ Dietary patterns rich in fish consumption may be protective for CRC and a study of the UKWCS concludes that women adhering to a Mediterranean dietary pattern also low in red meat may have a lower risk of CRC, especially rectal cancer.^29^ In our study, risk estimates for rectal cancer, showed a weak protective association in the case of fish‐eaters and vegetarians, with a null association in the poultry eaters group. However, none of the results reached statistical significance.

The effect of poultry on CRC in not clear. A meta‐analysis studying meat subtypes found no association for poultry consumption and risk of CRC.^40^ Results regarding poultry eaters in our study is in concordance with this, although there is a suggestion of a non‐significant protective effect on colon cancer. However, due to the low number of cases in the poultry group all results should be interpreted with care. It is interesting to note that the poultry eating dietary pattern in our study was characterized by consumption of similar amounts of fish as were consumed in the fish eating pattern. No further sub‐analysis of poultry eaters (with and without eating fish) was carried out due to the low number of participants in this group.

### Exploratory colon cancer subsite analysis

Exploratory analysis of colon subsites showed that grouped meat‐free diets showed a significant negative association with risk of distal colon cancer compared to red meat diets. Only a limited number of prospective studies have looked at the relationship between meat or dietary patterns containing meat and development of CRC by subsite across the colon (i.e., proximal *vs*. distal colon).^4^, ^5^, ^41^, ^42^, ^43^ Our findings appear to be consistent with previous studies where high levels of red meat were associated with distal colon cancer.^9^ However, previous research has also reported that red meat may be more strongly associated with colorectal and colon cancers but not with rectal cancer^4^ while processed meat may be more strongly associated with distal cancers than proximal cancers.^43^ Other studies have seen no association in all three subsites.^44^ Our cohort consumes low intakes of processed red meat and therefore we might expect lower numbers of cases of distal cancers.

There are some biological explanations that support the risk of red and processed meat on the distal colon. The concentrations of the pro‐mutagenic lesion O6‐methyldeoxyguanosine, a marker of exposure to many NOCs, have been shown to be significantly greater in tissues from the distal colon and rectum than from the proximal colon.^45^ However, further research is needed to clarify this point. A further explanation could be that butyrate concentrations are highest in the distal colon. Butyrate is produced by fermentation of dietary fiber and has been shown to induce apoptosis and to be cytotoxic to colorectal adenoma cells.^41^

The proximal colon and distal colon arise from different embryonic tissues, serve different functions, mucosal properties and microenvironment differ between segments, and are exposed to fecal matter for different durations of time. Hence, it has been suggested that the proximal and distal colon should be considered separately in aetiological studies of cancer, with the splenic flexure as a demarcation point.^46^ However, other studies challenge the current two‐colon paradigm and suggest that the frequencies of key tumor molecular features change gradually along the length of the colon. As meat consumption may impact differently across the three regions of the colorectum (proximal colon, distal colon or rectum), differences by types of red meat and by dietary patterns and cancer location is one of the biggest challenges in the study of diet and CRC with true associations remaining unclear.

### Strengths

Our study has several important strengths: the UKWCS is a large cohort with varied dietary intakes and a long follow up period and this is one of the largest analysis on this topic in the UK. The population‐based design enhances the generalizability of our results; specific subsites within the colon were examined separately. In addition, because exposure information was collected before the cancer diagnosis, any measurement error would have been non differential between cases and non‐cases and would most likely weaken any true association rather than causing an overestimation.

### Limitations

Since this is a prospective study, risk of recall bias is reduced. However, an ongoing challenge in nutritional epidemiology is accurate measurement of food intake. The FFQ used in this cohort has been validated against biomarkers^37^ and follows recommendations for good design.^38^ Our cohort is generally healthy as evidenced by relatively low smoking rates and low body mass index.^11^ It is therefore possible that less healthy dietary patterns were underrepresented in our cohort making differences between groups harder to elicit. The pragmatic definition of the dietary patterns used in this analysis may have led to the non‐significant findings. Use of categories in this way to define dietary patterns does not allow for examination of a possible dose–response effect of key components of the diet. In this analysis, processed meat was included as red meat. Colon cancer subsite analysis is presented as exploratory due to the limited power and multiple comparisons. Only women were included in our study but there is no clear evidence around variation between men and women in previous research.^17^, ^45^

## Conclusion

In summary, grouped and independently analyzed red‐meat free diets showed a non‐significantly decreased risk of CRC compared to red meat eaters. Only exploratory subsite analysis showed a significant risk reduction for distal colon cancer in red meat‐free dietary patterns. These results indicate that protective associations of red meat free diets on colorectal cancers merit further investigation in a larger study with larger numbers of cases.
